# Birth weight and large for gestational age trends in offspring of pregnant women with gestational diabetes mellitus in southern China, 2012-2021

**DOI:** 10.3389/fendo.2023.1166533

**Published:** 2023-05-04

**Authors:** Li-Rong He, Li Yu, Yong Guo

**Affiliations:** ^1^ Department of Obstetrics, Guangdong Women and Children Hospital, Guangzhou Medical University, Guangzhou, China; ^2^ Department of Children’s Health Care, Guangdong Women and Children Hospital, Guangzhou Medical University, Guangzhou, China

**Keywords:** birth weight, gestational diabetes mellitus (GDM), large for gestational age (LGA), trends, pregnancy

## Abstract

**Background:**

With increasing prevalence of gestational diabetes mellitus (GDM) and changing management of GDM in pregnancy, it is imperative to understand the evolution of its current outcomes. The present study aimed to explore whether birth weight and large for gestational age (LGA) trends in women with GDM have changed over time in southern China.

**Methods:**

In this hospital-based retrospective study, all singleton live births for the period 2012 to 2021 were collected from the Guangdong Women and Children Hospital, China. GDM was diagnosed following the criteria of the International Association of Diabetes and Pregnancy Study Group. The cutoff points for defining LGA (>90th centile) at birth based on INTERGROWTH-21st gender-specific standards. Linear regression was used to evaluate trends for birth weight over the years. Logistic regression analysis was used to determine the odds ratios (ORs) of LGA between women with GDM and those without GDM.

**Results:**

Data from 115097 women with singleton live births were included. The total prevalence of GDM was 16.8%. GDM prevalence varied across different years, with the lowest prevalence in 2014 (15.0%) and the highest prevalence in 2021 (19.2%). The mean birth weight displayed decrease in women with GDM from 3.224kg in 2012 to 3.134kg in 2021, and the z score for mean birth weight decreased from 0.230 to -0.037 (P for trend < 0.001). Among women with GDM, the prevalence of macrosomia and LGA reduced significantly during the study period (from 5.1% to 3.0% in macrosomia and from 11.8% to 7.7% in LGA, respectively). Compared to women without GDM, women with GDM had 1.30 (95% CI: 1.23 - 1.38) times odds for LGA, and the ORs remained stable over the study period.

**Conclusions:**

Among offspring of women with GDM, there are decreased trends of birth weight in parallel with reductions in LGA prevalence between 2012 and 2021. However, the risk of LGA in women with GDM remains stable at relatively high level over the 10-year period, and efforts are still needed to address regarding causes and effective intervention strategies.

## Introduction

Birth weight is an important predictor of neonatal morbidity and mortality, reflecting both maternal health and neonatal health (1, 2). In the recent past, a number of researchers have demonstrated the trends in birth weight. Data from the United States and the United Kingdom showed an increasing trend in mean birth weight (3, 4). Increased birth weight is associated with early neonatal complications, as well as cardiovascular and metabolic disease later in adulthood (5). Around 50% of pregnancy women with pre-existing diabetes mellitus are delivering large for gestational age (LGA) neonates (6, 7). The emergence of new technologies for managing diabetes mellitus is revolutionizing the management of adverse conditions in pregnancy. However, several studies have observed a paradoxical trend that infant born to women with type 1 diabetes increasingly show overgrowth despite apparent good maternal glycemic control (8). Pregnancy women with diabetes are receiving increased intervention in Scotland, but a continuous increase in birth weight and the proportion of LGA were found from 1998 to 2013 (9). Fetal macrosomia and LGA infants born to women with diabetes were increased between 1991 and 2003 in Sweden (10) and between 1987 and 2016 in Australia (11). Gestational diabetes mellitus (GDM), which is the most common complication during pregnancy, is a definitive risk factor for fetal overgrowth and long-term offspring complications (12–14). The prevalence of GDM is increasing worldwide during the past few decades (15). In 2011, after the new diagnostic criteria of the International Association of Diabetes and Pregnancy Study Group (IADPSG) was gradually carried out, the prevalence of GDM increased almost 3-5 times, up to 14.8% in mainland China (16). Despite marked improvement in managing blood glucose levels, women with GDM still carries risks for the growing fetus. It is not clear whether birth weight and the proportion of LGA in offspring of women, who are diagnosed as GDM by the IADPSG criteria, have changed over time in China. Understanding the past and current trends of the birth weight and LGA is imperative to improving the health outcomes for women with GDM. The present study aimed to examine the 10-year trends in birth weight and prevalence of LGA between women with and without GDM, using hospital-based databases (2012–2021) in southern China.

## Methods

### Study population

This was a hospital-based retrospective study, which was conducted in Guangdong Women and Children Hospital, the provincial health center for maternal and child health surveillance of Guangdong, southern China. All singleton pregnancies with live birth between January 2012 and December 2021 were retrospectively selected from the hospital information system. Live births with gestational age between 24 and 42 weeks were included. For mothers who had GDM screening and delivered in Guangdong Women and Children Hospital were included. The data (maternal age, parity, mode of delivery, date of newborn’s birth, gestational week at birth, and birth weight) was collected from the electronic medical records of Guangdong Women and Children Hospital. We excluded women with hypertensive disorders, pre-pregnancy diabetes, multiple pregnancies or fetal anomalies or missing data on gestational age.

### Ethical statement

Ethical approval was obtained from the Ethics Committee of the Guangdong Women and Children Hospital. In accordance with national legislation and institutional regulations, written informed consent for participation was not necessary for this study. The accessed patient data adhered to applicable data protection and privacy regulations.

### Screening and diagnoses of GDM

During the study period, pregnant women were screened for GDM using IADPSG criteria at 24-28 weeks of gestation. GDM was diagnosed if any of the blood glucose values equals to or exceeds: fasting blood glucose 5.1 mmol/L, 1-h blood glucose 10.0 mmol/L, and 2-h blood glucose 8.5 mmol/L (17).

### Statistical analysis

We defined low birth weight as a birth weight of <2.5 kg, and macrosomia as a birth weight ≥ 4.0 kg. Birth weight was also calculated as birth weight z-scores using the INTERGROWTH-21st standards (18). The cutoff points for defining small for gestational age (<10th centile) and LGA (>90th centile) at birth based on INTERGROWTH-21st gender-specific standards.

Continuous variables were reported as the mean ± standard deviation, and categorical variables were reported as numbers and percentages. To compare differences between women with GDM and those without GDM, the t-test was used for continuous variables and the chi-square test was used for categorical variables. Linear regression was used to evaluate trends for birth weight over the years. The annual percentage change (APC) of trends were determined based on logarithmically transformed percentages and their standard errors. Logistic regression analysis was used to determine the odds ratios (ORs) of the macrosomia and LGA between women with GDM and those without GDM, adjusting for maternal age and parity. Data analysis was performed using the SPSS statistical software package (V26, IBM Statistics, Chicago, IL, USA). P <0.05 was considered to be the threshold for statistical significance in analyses.

## Results

**Figure 1:**
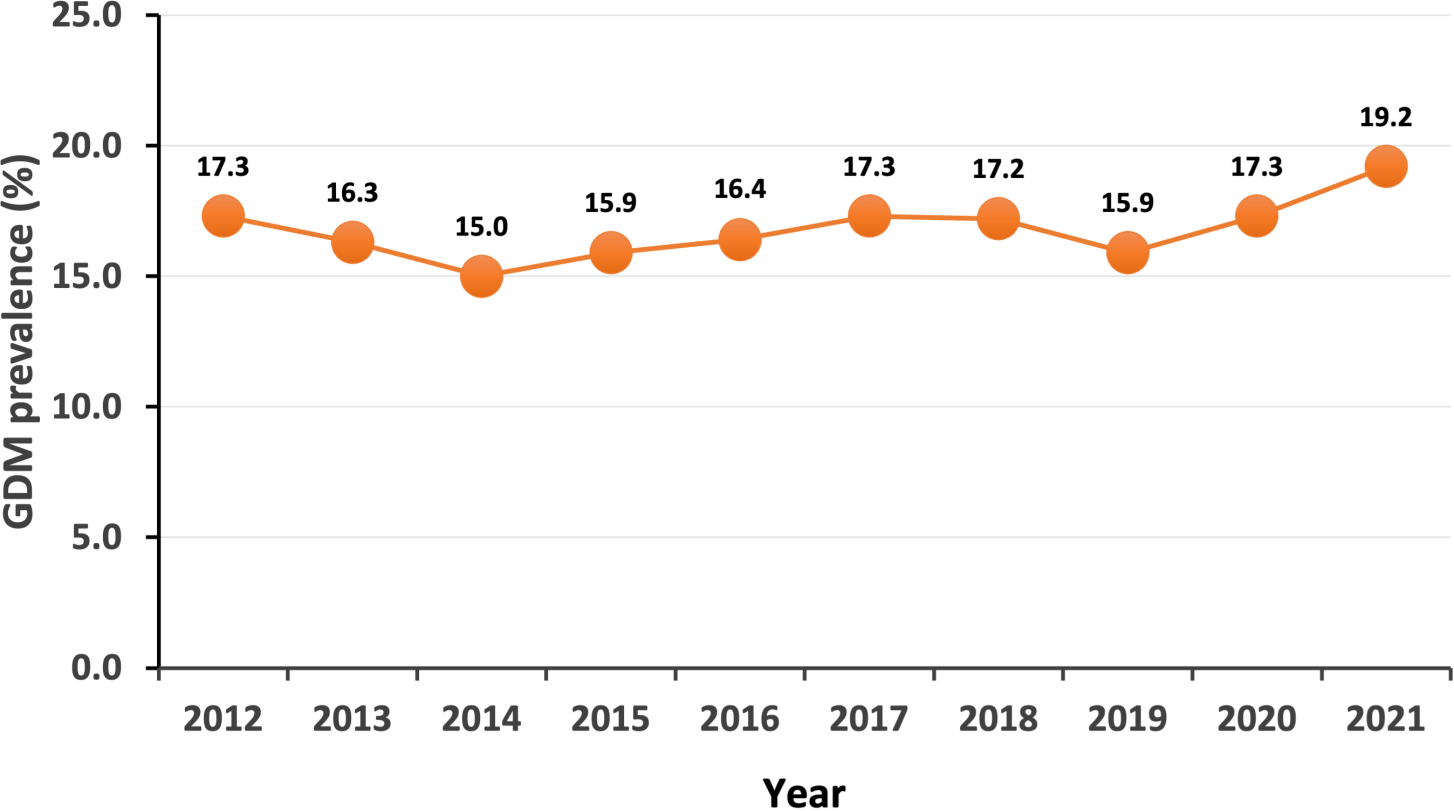
Trends of prevalence of GDM between 2012 and 2021.

A total of 115097 women with singleton live births were included in the study. The prevalence of GDM was 16.8%, which varied across different years, with the lowest prevalence in 2014 (15.0%) and the highest prevalence in 2021 (19.2%) (**Figure 1**). There were four significant trend periods for prevalence of GDM during this period: decreased from 2012 to 2014 with an APC of -6.9, increased from 2014 to 2017 with an APC of 4.7, decreased from 2017 to 2019 with an APC of -4.1, increased from 2019 to 2021 with an APC of 9.8. **Table 1** shows the characteristics of the participants. Compared to the women without GDM, the women with GDM had higher maternal age, higher proportions of multiparous and cesarean. The prevalence of macrosomia and LGA were significantly higher in women with GDM than those without GDM.

**Table 1:** Characteristics of the study population.

Variables	Total (n= 115097)	Non-GDM (n= 95741)	GDM (n= 19356)	*P*-value
Maternal age at delivery, years	29.7 ± 4.6	29.4 ± 4.5	31.7 ± 4.8	< 0.001
18-24	13643 (11.9)	12620 (13.2)	1023 (5.3)	< 0.001
25-29	45617 (39.6)	39950 (41.7)	5667 (29.3)	
30-34	37929 (33.0)	30600 (32.0)	7329 (37.9)	
35-39	14746 (12.8)	10588 (11.1)	4158 (21.5)	
>=40	3162 (2.7)	1983 (2.1)	1179 (6.1)	
Parity				
Nulliparous	58582 (50.9)	50040 (52.3)	8542 (44.1)	< 0.001
econd delivery	47359 (41.1)	38488 (40.2)	8871 (45.8)	
Third and more delivery	9156 (8.0)	7213 (7.5)	1943 (10.0)	
Mode of delivery				
Vaginal	71953 (62.5)	60990 (63.7)	10963 (56.6)	
Cesarean	43144 (37.5)	34751 (36.3)	8393 (43.4)	
Gestation at delivery, weeks	38.99 ± 1.86	39.04 ± 1.85	38.75 ± 1.89	< 0.001
24-31	1689 (1.5)	1361 (1.4)	328 (1.7)	< 0.001
32-36	7249 (6.3)	5678 (5.9)	1571 (8.1)	
37-38	33691 (29.3)	27427 (28.6)	6264 (32.4)	
39-40	65004 (56.5)	54304 (56.7)	10700 (55.3)	
41-42	7464 (6.5)	6971 (7.3)	493 (2.5)	
Newborn sex				
Male	61706 (53.6)	51131 (53.4)	10575 (54.6)	0.002
Female	53391 (46.4)	44610 (46.6)	8781 (45.4)	
Birth weight, kg	3.171 ± 0.497	3.172 ± 0.491	3.167 ± 0.525	0.148
Low birth weight (<2.5 kg)	7767 (6.7)	6243 (6.5)	1524 (7.9)	< 0.001
Macrosomia (≥ 4.0 kg)	3789 (3.3)	2962 (3.1)	827 (4.3)	
Z score for birthweight	0.001 ± 0.883	-0.014 ± 0.873	0.078 ± 0.928	< 0.001
Small for gestational age (≤ 10th centile)	8039 (7.0)	6797 (7.1)	1242 (6.4)	< 0.001
Large for gestational age (≥ 90th centile)	8530 (7.4)	6630 (6.9)	1900 (9.8)	

Values are presented as n (%) or mean ± SD.

*P*-value for comparing variable between Non-GDM and GDM group.

**Figure 2:**
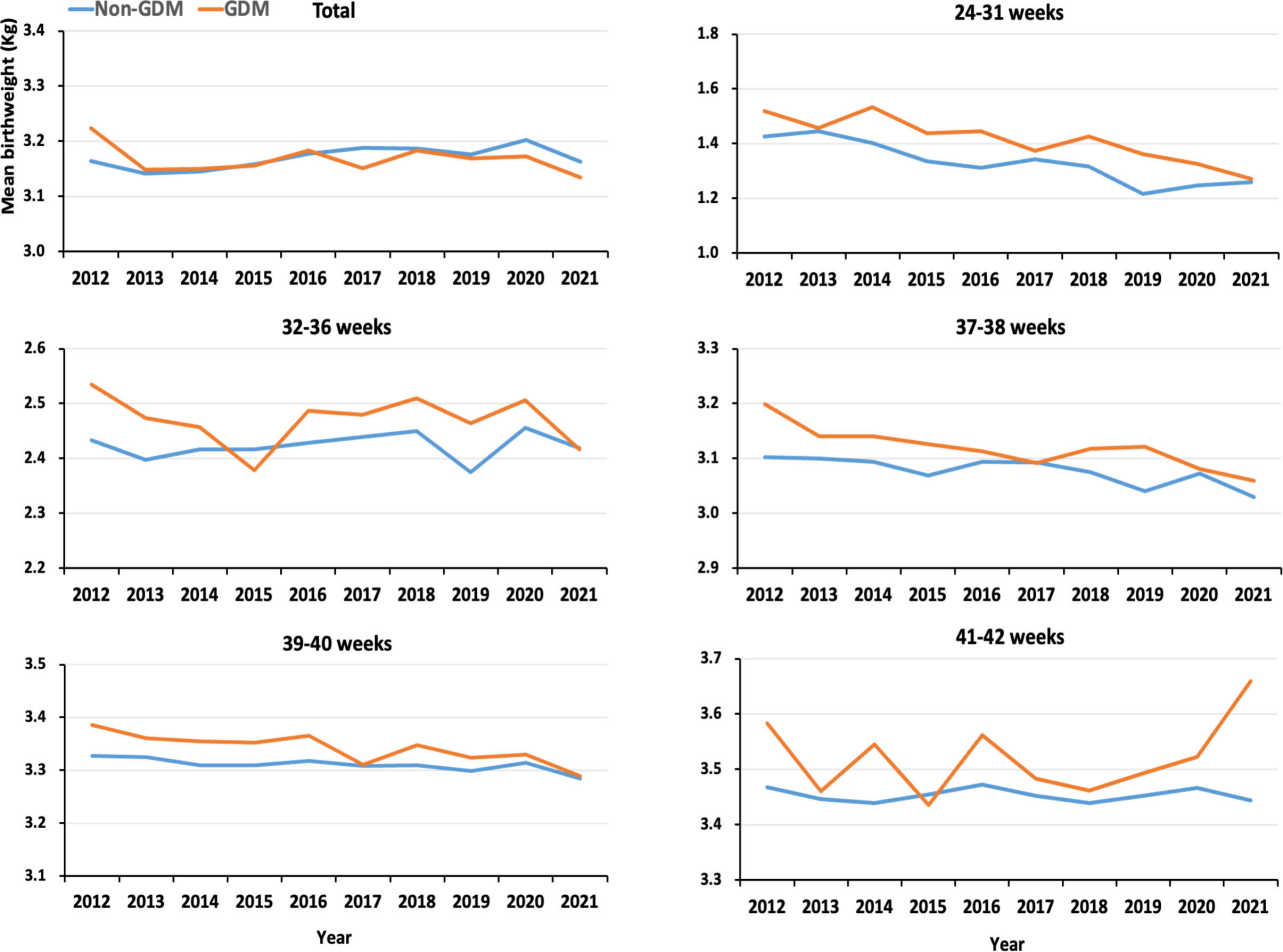
Trends of mean birth weight in women with and without GDM between 2012 and 2021.

**Figure 2** shows the trends of mean birthweight in women with and without GDM between 2012 and 2021. The mean birth weight appeared almost flat trend for the 10-year period in women without GDM, but displayed decrease in women with GDM from 3.224kg in 2012 to 3.134kg in 2021. Decreased changes of mean birth weight over times were significant for groups 24-31 weeks, 37-38 weeks, and 39-40 weeks gestational age at delivery in women with GDM (**Figure 2**). The results appeared to be no significant change in the absolute values of birth mean weight between offspring of women with GDM and those without GDM, although there was a statistically significant difference due to the large sample size. However, the z-score for birth weight showed that GDM offspring had higher birth weight than non-GDM offspring, and both groups demonstrated a decreasing trend (**Table 2**). When we further restricted the analysis to full-term singleton live births, similar results were observed (**Supplementary Table 1**).

**Table 2:** Trends in singleton liveborn birth weight in women with and without GDM between 2012 and 2021.

Year	Total No.	GDM No.	Birth weight (kg)		*P*-value	Birth weight z score		*P*-value
			Non-GDM	GDM		Non-GDM	GDM	
2012	8920	1539	3.164 ± 0.514	3.224 ± 0.504	< 0.001	0.039 ± 0.904	0.230 ± 0.905	< 0.001
2013	8637	1407	3.141 ± 0.530	3.149 ± 0.561	0.606	0.031 ± 0.884	0.144 ± 0.946	< 0.001
2014	10003	1501	3.145 ± 0.526	3.150 ± 0.574	0.757	-0.004 ± 0.891	0.112 ± 0.972	< 0.001
2015	10400	1650	3.158 ± 0.522	3.156 ± 0.565	0.884	-0.024 ± 0.892	0.078 ± 0.971	< 0.001
2016	11918	1951	3.177 ± 0.503	3.183 ± 0.528	0.641	0.018 ± 0.879	0.133 ± 0.913	< 0.001
2017	13819	2394	3.188 ± 0.474	3.151 ± 0.509	0.001	-0.001 ± 0.866	0.023 ± 0.905	0.232
2018	13012	2239	3.187 ± 0.475	3.183 ± 0.529	0.763	-0.019 ± 0.861	0.088 ± 0.944	< 0.001
2019	14113	2240	3.176 ± 0.469	3.169 ± 0.517	0.546	-0.06 ± 0.854	0.059 ± 0.920	< 0.001
2020	12193	2111	3.202 ± 0.459	3.173 ± 0.503	0.010	-0.006 ± 0.861	0.043 ± 0.926	0.019
2021	12082	2324	3.163 ± 0.459	3.134 ± 0.490	0.006	-0.085 ± 0.850	-0.037 ± 0.885	0.015
*P*-value for trend			0.045	0.019		0.027	< 0.001	

**Figure 3:**
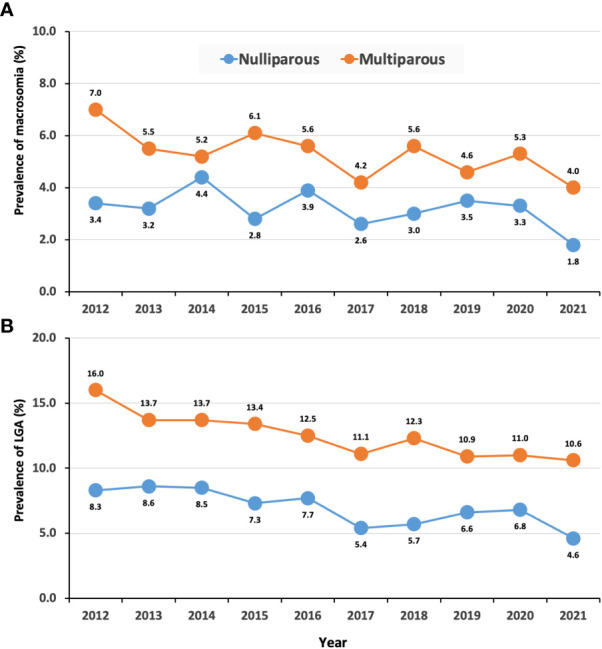
Trends in prevalence of macrosomia and LGA in women with GDM by parity. **(A)**, macrosomia; **(B)**, LGA.

Among women with GDM, the prevalence of macrosomia and LGA reduced significantly during the study period (from 5.1% to 3.0% in macrosomia and from 11.8% to 7.7% in LGA, respectively) (**Table 3**). The prevalence of macrosomia and LGA in offspring of women with GDM was higher than that of non-GDM women. Despite fluctuations in the prevalence of macrosomia over the past 10 years, the prevalence of macrosomia did not significantly decrease in non-GDM offspring, while it showed a relatively significant reduction in GDM offspring. The prevalence of LGA showed a decreasing trend in both groups (**Table 3**). **Figure 3** revealed that prevalence of macrosomia and LGA was significantly higher in multiparous women with GDM than those nulliparous women across the time course examined. The prevalence of macrosomia and LGA showed decreased trends with time in both nulliparous and multiparous. Women with GDM were risk factors for macrosomia and LGA. Compared to women without GDM, women with GDM had 1.30 (95% CI: 1.23 - 1.38) times odds for LGA, and the odds remained stable over the study years (**Table 4**).

**Table 3:** Trends of prevalence of singleton liveborn macrosomia and LGA in women with and without GDM between 2012 and 2021.

Year	Macrosomia, prevalence (95% CI)		*P*-value	Large for gestational age, prevalence (95% CI)		*P*-value
	Non-GDM	GDM		Non-GDM	GDM	
2012	3.4 (3.0 - 3.8)	5.1 (4.1 - 6.2)	0.002	8.2 (7.6 - 8.9)	11.8 (10.3 - 13.5)	< 0.001
2013	3.3 (2.9 - 3.7)	4.3 (3.4 - 5.5)	0.041	7.8 (7.2 - 8.5)	11.2 (9.7 - 13.0)	< 0.001
2014	3.0 (2.7 - 3.4)	4.8 (3.8 - 6.0)	< 0.001	7.1 (6.6 - 7.6)	11.2 (9.7 - 12.9)	< 0.001
2015	3.0 (2.7 - 3.4)	4.6 (3.7 - 5.7)	0.001	7.1 (6.6 - 7.7)	10.7 (9.2 - 12.2)	< 0.001
2016	3.4 (3.1 - 3.8)	4.9 (4.0 - 5.9)	0.002	7.4 (6.9 - 8.0)	10.4 (9.1 - 11.8)	< 0.001
2017	3.4 (3.1 - 3.8)	3.6 (2.9 - 4.4)	0.663	7.1 (6.6 - 7.5)	9.1 (8.0 - 10.3)	0.001
2018	3.0 (2.7 - 3.3)	4.6 (3.8 - 5.5)	< 0.001	6.9 (6.4 - 7.3)	9.8 (8.6 - 11.1)	< 0.001
2019	2.7 (2.4 - 3.0)	4.2 (3.4 - 5.0)	< 0.001	5.9 (5.5 - 6.4)	9.1 (8.0 - 10.4)	< 0.001
2020	3.5 (3.2 - 3.9)	4.5 (3.6 - 5.4)	0.034	6.9 (6.4 - 7.4)	9.2 (8.0 - 10.5)	< 0.001
2021	2.4 (2.1 - 2.7)	3.0 (2.3 - 3.7)	0.136	5.6 (5.1 - 6.0)	7.7 (6.7 - 8.8)	< 0.001
*P*-value for trend	0.665	0.030		0.009	< 0.001	

**Table 4:** Trends in odds ratio of macrosomia and LGA in women with GDM.

Year	Macrosomia			Large for gestational age		
	OR	95% CI	*P*-value	OR	95% CI	*P*-value
2012	1.40	1.07 - 1.82	0.014	1.36	1.14 - 1.63	0.001
2013	1.20	0.90 - 1.61	0.221	1.34	1.10 - 1.62	0.003
2014	1.45	1.10 - 1.91	0.008	1.46	1.21 - 1.76	< 0.001
2015	1.34	1.02 - 1.75	0.034	1.33	1.11 - 1.60	0.002
2016	1.31	1.03 - 1.66	0.029	1.24	1.05 - 1.47	0.012
2017	0.95	0.75 - 1.22	0.701	1.16	0.99 - 1.36	0.071
2018	1.39	1.10 - 1.76	0.005	1.28	1.08 - 1.50	0.003
2019	1.43	1.12 - 1.82	0.004	1.39	1.17 - 1.64	< 0.001
2020	1.19	0.94 - 1.51	0.149	1.22	1.03 - 1.44	0.024
2021	1.20	0.91 - 1.59	0.192	1.33	1.11 - 1.59	0.002
Total	1.27	1.17 - 1.38	< 0.001	1.30	1.23 - 1.38	< 0.001

OR were adjusted for maternal age and parity.

## Discussion

GDM is becoming more prevalent, and it is imperative to gain a better understanding of the evolution of adverse outcomes. In the management of pregnancy complicated by GDM, birth weight and LGA are important outcome measures. Therefore, our study updated the 10-year trends in the birth weight and LGA among women with GDM in southern China. This large, hospital‐based study documented a high prevalence of GDM with fluctuations over time. The birth weight appeared decrease trends in women with GDM, and also found a concomitant decline in LGA prevalence from 2012 to 2021.

The overall prevalence of GDM was 16.8% during the 10‐year study period, which was contrast with those of a previous report evaluating trends in GDM prevalence in China (16) and other parts of the world (19). A recent clinical update on GDM by Sweeting et al. also highlighted the increasing prevalence of GDM globally, with estimates suggesting that GDM affects around 2% to 19% for the IADPSG criteria of all pregnancies worldwide, and emphasized the importance of appropriate management of blood glucose levels in preventing adverse outcomes in pregnancies complicated by GDM (20). GDM affects 17.6% of all pregnant women from 2011 to 2018, with high and stable trend in the prevalence of GDM in Xiamen, China (21). Another study conducted in Beijing during 2013-2018 reported that the prevalence of GDM was 24.2% according to the IADPSG criteria (22). After applying the IADPSG criteria in China, the prevalence of GDM was substantially increased due to more pregnant women with mild hyperglycemia were diagnosed as GDM. Although these studies conducted to be in line with the same IADPSG criteria in China, there were some deficiencies in these previous studies, which could be partly relevant to China with a large population in different regions, ethnicities, diets, and living habits (16).

Although multiple studies had evaluated GDM prevalence in China at some point, few examined trends of birth weight and LGA prevalence for 10-year period after employing the new criteria. Our study showed that birth weight was decreased in women with GDM during this study period. Trends in the prevalence of macrosomia (birth weight ≥ 4.0 kg) and LGA were declined throughout the study period in women with GDM. Positive changes over this time period, such as improved antenatal care and progressed in managing blood glucose levels, may be contributed to the decreasing trends in LGA. This finding is contrary to previous study surveyed in the UK which have reported that average birth weight is greatly increased in the offspring of mothers with diabetes, despite receiving increased intervention in pregnancy between 1998 and 2013 (9). It may be interpreted as numbers of pregnancy women with type 1 and type 2 diabetes have increased significantly over that study period (23). The prevalence of LGA decreased during our study period, but the risk for LGA in women with GDM was found no significant change. The metabolic abnormalities of GDM during pregnancy are mainly due to increased insulin resistance and β-cell defects (24), which most commonly involve hyperglycemia with the attendant risk of fetal overgrowth (25). However, women with GDM often have other risk factors for LGA, including increasing age, maternal overweight, excessive gestational weight gain and insulin administration (26–28). Our present study did not investigate the comprehensive effects of GDM. Thus, the findings could not be determined whether in relation to risk factors for LGA associated with GDM were due to maternal hyperglycemia or other risk factors.

Our study is mainly limited by its retrospective design. We used routinely collected data in hospital information system that were unable to measure some factors assessed in the previous studies, such as maternal gestational weight gain, antenatal care and glycemic control status during pregnancy across the study period. Second, the study was conducted using non-random population-based sampling. The data may have been affected by selection bias, compromising its representativeness. However, continued monitoring of recent trends is needed to assess improvement made in reducing pregnancies complicated by GDM. It is also important for future studies to analyze the tendency of offspring birth weight in women with GDM over time, as well as exploring the causes. In addition, it is important to consider that diagnostic criteria for GDM may vary across different countries and racial/ethnic groups. The IADPSG criteria, which we used in our study, have been adopted by many countries, but other countries may use different criteria that could affect the reported prevalence of GDM (29). Our study was conducted in southern China where the population is predominantly Han Chinese, it is known that different racial/ethnic groups have different risks of developing and managing GDM (30). Studies have shown that South Asian and Hispanic women are at higher risk of developing GDM compared to non-Hispanic white women (31, 32). This may be attributed to differences in genetics, lifestyle factors, and socioeconomic status (33). Therefore, future studies should investigate the trends in birth weight and LGA in different racial/ethnic groups with GDM and compare outcomes using the same diagnostic criteria. This could help identify any disparities in the management and outcomes of GDM in different populations and inform the development of tailored interventions to improve maternal and fetal health.

## Conclusions

Our study observed that there are decreased trends of birth weight in women with GDM and a concomitant decline in LGA prevalence between 2012 and 2021. Although these results partly represent improvements in avoiding fetal overgrowth for women with GDM over the 10-year period, the risk of LGA in women with GDM remains at relatively high level, and efforts are still needed to address regarding causes and effective interventions for adverse outcomes.

## Data availability statement

The raw data supporting the conclusions of this article will be made available by the authors, without undue reservation.

## Ethics statement

The studies involving human participants were reviewed and approved by Ethics Committee of the Guangdong Women and Children Hospital. Written informed consent for participation was not required for this study in accordance with the national legislation and the institutional requirements.

## Author contributions

Conception and design of this study: L-RH, LY and YG. Data collection and analysis: L-RH and YG. The first draft of the manuscript was written by YG and all authors commented on previous versions of the manuscript. All authors contributed to the article and approved the submitted version.
