# Supplementary material for: Birth weight and large for gestational age trends in offspring of pregnant women with gestational diabetes mellitus in southern China, 2012-2021
Source: Front Endocrinol (Lausanne). 2023 May 4;14:1166533. doi: 10.3389/fendo.2023.1166533 (PMC10194652; doi:10.3389/fendo.2023.1166533)
Supplement: Supplementary file 1 [file DataSheet_1.pdf]

Supplementary Table 1 Trends in full-term singleton liveborn birth weight in women with and without GDM between 2012 and 2021

Year	Total No.	GDM No.	Birth weight (kg)		*P*-value	Birth weight z score		*P*-value
			Non-GDM	GDM		Non-GDM	GDM	
2012	8071	1375	3.257 ± 0.402	3.319 ± 0.405	< 0.001	0.052 ± 0.902	0.253 ± 0.899	< 0.001
2013	7694	1225	3.252 ± 0.395	3.278 ± 0.422	0.042	0.041 ± 0.881	0.150 ± 0.950	< 0.001
2014	8983	1300	3.249 ± 0.392	3.282 ± 0.440	0.006	0.002 ± 0.887	0.132 ± 0.967	< 0.001
2015	9461	1468	3.252 ± 0.397	3.275 ± 0.434	0.041	-0.016 ± 0.888	0.091 ± 0.974	< 0.001
2016	10959	1756	3.259 ± 0.393	3.279 ± 0.418	0.047	0.030 ± 0.879	0.137 ± 0.912	< 0.001
2017	12820	2162	3.255 ± 0.387	3.244 ± 0.398	0.23	0.005 ± 0.867	0.033 ± 0.910	0.183
2018	12130	2036	3.252 ± 0.384	3.272 ± 0.420	0.031	-0.014 ± 0.863	0.095 ± 0.944	< 0.001
2019	13233	2049	3.237 ± 0.382	3.256 ± 0.408	0.043	-0.046 ± 0.852	0.065 ± 0.919	< 0.001
2020	11473	1942	3.255 ± 0.387	3.246 ± 0.416	0.336	0.002 ± 0.861	0.047 ± 0.924	0.038
2021	11335	2144	3.220 ± 0.383	3.211 ± 0.391	0.333	-0.076 ± 0.848	-0.030 ± 0.887	0.023
P-value for trend			0.075	0.001		0.006	0.001	

Supplementary Table 2 Trends of prevalence of full-term singleton liveborn macrosomia and LGA in women with and without GDM between 2012 and 2021

Year	Macrosomia, prevalence (95% CI)		*P*-value	Large for gestational age, prevalence (95% CI)		*P*-value
	Non-GDM	GDM		Non-GDM	GDM	
2012	3.8 (3.3 - 4.2)	5.7 (4.5 – 7.0)	0.001	8.6 (7.9 - 9.3)	12.3 (10.6 - 14.1)	< 0.001
2013	3.6 (3.2 - 4.1)	5.0 (3.9 - 6.3)	0.025	8.1 (7.4 - 8.8)	12.0 (10.3 - 13.9)	< 0.001
2014	3.3 (2.9 - 3.7)	5.5 (4.3 - 6.8)	< 0.001	7.4 (6.8 – 8.0)	11.5 (9.8 - 13.3)	< 0.001
2015	3.3 (2.9 - 3.7)	5.2 (4.1 - 6.4)	< 0.001	7.3 (6.7 - 7.9)	11.2 (9.6 - 12.9)	< 0.001
2016	3.7 (3.3 - 4.1)	5.2 (4.3 - 6.4)	0.002	7.8 (7.3 - 8.4)	10.8 (9.4 - 12.3)	< 0.001
2017	3.7 (3.3 – 4.0)	3.9 (3.2 - 4.8)	0.541	7.2 (6.8 - 7.7)	9.4 (8.3 - 10.7)	< 0.001
2018	3.2 (2.8 - 3.5)	5.0 (4.1 – 6.0)	< 0.001	7.1 (6.6 - 7.6)	10 (8.8 - 11.4)	< 0.001
2019	2.8 (2.5 - 3.1)	4.5 (3.7 - 5.5)	< 0.001	6.2 (5.7 - 6.6)	9.2 (8.0 - 10.5)	< 0.001
2020	3.7 (3.3 - 4.1)	4.7 (3.9 - 5.8)	0.032	7.1 (6.6 - 7.6)	9.3 (8.1 - 10.7)	0.001
2021	2.6 (2.3 - 2.9)	3.2 (2.5 – 4.0)	0.100	5.7 (5.3 - 6.2)	7.9 (6.8 - 9.1)	< 0.001
P-value for trend	0.082	0.001		0.010	< 0.001	
